# Gut Microbiota Feature of Senile Osteoporosis by Shallow Shotgun Sequencing Using Aged Rats Model

**DOI:** 10.3390/genes13040619

**Published:** 2022-03-29

**Authors:** Ning Wang, Sicong Ma, Lingjie Fu

**Affiliations:** Shanghai Key Laboratory of Orthopaedic Implants, Department of Orthopaedic Surgery, Shanghai Ninth People’s Hospital, Shanghai Jiao Tong University School of Medicine, Shanghai 200011, China; wangn2012@126.com (N.W.); thatrobynn@163.com (S.M.)

**Keywords:** senile osteoporosis, gut microbiota, bacteria species, shallow shotgun, functional metabolic pathway

## Abstract

Senile osteoporosis is defined as an age-related bone metabolic disorder, which is characterized by bone loss and decreased bone fragility. Gut microbiota (GM) could regulate the bone metabolic process and be closely related to senile osteoporosis. Several genus-level GM were found to increase in osteoporotic animals and patients. However, to reveal the pathogenic bacteria in senile osteoporosis, further studies are still needed to investigate the complete characteristics of bacteria species. In the present study, the rats were equally divided into two groups: the control group (Con, 6-month-old) and the osteoporosis group (OP, 22-month-old). Fecal samples were freshly collected to conduct the shallow shotgun sequencing. Then, we compared the species numbers, microbial diversity, GM composition at genus and species-level, and functional metabolic pathways in the two groups. The results showed that the species number was lower in the OP group (1272) than in the control group (1413), and 1002 GM species were shared between the two groups. The OP group had the decreased α diversity compared with the control group. As for β diversity, The PCA revealed that samples in the two groups had distinguishable ecological distance in each coordinate. At the species level, *Bacteroide coprocola (B. coprocola)*, *Acinetobacter baumannii (A. baumannii)*, *Parabacteroides distasonis (P. distasonis)*, and *Prevotella copri (P. copri)* were higher in the OP group, while *Corynebacterium stationis (C. stationis)*, *Akkermansia muciniphila (A. muciniphila)*, and *Alistipes indistinctus (A. indistinctus)* were decreased. Moreover, functional metabolic analysis revealed that metabolic pathways of fatty acid biosynthesis, valine/isoleucine biosynthesis, GABA biosynthesis, and ubiquinone biosynthesis were enriched in the senile osteoporotic rats. In conclusion, GM at the species level in senile osteoporotic rats was significantly altered in structure, composition, and function. The altered GM structure, increased GM species such as *P. copri*, and decreased GM species such as *A. muciniphila* might be linked with the development of senile osteoporosis.

## 1. Introduction

Gut microbiota (GM) are defined as the collection of commensal bacteria living in the digestive tract, which regulates host metabolism and performs various functions. GM of humans consist of over 1000 distinct bacterial species, about two-thirds of which are unique to each individual [1]. GM have an impact on many chronic diseases, such as obesity, diabetes, neurological disorders, inflammatory bowel disease (IBD), and cardiovascular disease [2,3,4,5,6]. As a member of the chronic disease category, osteoporosis is also associated with GM [7].

Senile osteoporosis is a primary, age-related bone metabolic disease, which is characterized by bone loss and decreased bone fragility [8]. Commonly, primary osteoporosis mainly affects postmenopausal females and aged males. Commonly, postmenopausal females have a higher incidence of fragility fractures, while aged people, especially aged males, have a higher rate of mortality related to osteoporosis [9]. Emerging evidence demonstrated that aging plays a critical role in the development of osteoporosis [10]. With the gradual rise of an aging population, senile osteoporosis has brought a higher burden on the public health system [11].

Recent studies suggested that senile osteoporosis was closely related to GM. Several clinical studies reported that the osteoporotic patients showed altered GM, richness, diversity, relative GM abundance, and functional metabolic pathways [12,13,14,15,16]. However, due to the distinct geographical location, sample size, gender distribution, and dietary habits of patients, these clinical studies have not reached a consistent conclusion on the regulation of GM alteration in osteoporotic people. In an animal study, senile osteoporotic rats showed decreased α diversity, altered β diversity, and increased relative abundance of *Helicobacter*, *Rothia*, *Clostridium IV*, *Alistipes*, and *H. rodentium* using 16S rRNA, and the whole metagenome sequencing (WMS) [17]. Furthermore, another study found that GM derived from feces of aged rats led to bone loss, and increased the genus-level relative abundance of *Romboutsia*, *Faecalibacterium*, and *Lachnospiraceae_incertae_sedis* in young rats [18]. On the contrary, GM derived from young rats could restore the bone mass of senile osteoporotic rats and decrease the genus-level relative abundance of *Helicobacter* and *Prevotella* [19]. GM features at the genus level of senile osteoporosis were revealed by 16S rRNA sequencing using rats model.

Shallow shotgun sequencing is a high-accuracy microbiological technology, which could annotate the microbial taxonomy to the species level [20,21]. In addition, it could reach nearly the same sequencing depth and accuracy as WMS technology with fewer data [22]. To date, studies based on 16S rRNA sequencing about GM and senile osteoporosis only revealed genus-level GM taxonomic features. Although some GM strains such as *H. rodentium* were found to increase in the osteoporotic rats [17], the complete species-level characteristics of GM were still unknown.

Our study aimed to provide further insights into the pathogenic bacteria, especially at the species level, in senile osteoporosis. In the present study, we compared the GM richness and diversity of senile osteoporotic rats and young rats, and analyzed the GM composition at the species levels. Furthermore, we analyzed the GM composition at the genus level to verify whether the alteration in GM species was in accordance with that in the GM genus. In addition, we revealed the functional metabolic pathways markedly expressed in the fecal samples of senile osteoporosis.

## 2. Materials and Methods

### 2.1. Experimental Design

The experiment was approved by the Ethical committee of experimental animal care in the Shanghai Ninth People’s Hospital (Grant no. SH9H-2019-A17-1). According to the previous study, we purchased 6 young (six-month-old) and 6 aged (22-month-old) female Sprague Dawley (SD) rats from Shanghai Slac Laboratory Animal Company (Slac., Shanghai, China, SCXK2012-0002). These rats were equally divided into the control group (Con, 6-month-old, *n* = 6) and the osteoporosis group (OP, 22-month-old, *n* = 6) [22]. All rats were raised under 12-h-light-dark photoperiod with 2/per cages in the SPF condition. We fed rats with a pelleted rodent diet and autoclaved water [23]. Fecal samples of the two groups were freshly collected from each rat and were frozen immediately before they were stored at −80 °C.

### 2.2. Bone Mineral Density Detection

After being sacrificed by cervical dislocation, Bilateral femurs of each rat were removed and collected. Then, we used dual-energy X-ray absorptiometry (DXA, Hologic Discovery A, Boston, MA, USA) to detect the bone mineral density.

### 2.3. HE Staining of Femoral Sections

All femurs were embedded in paraffin after they were fixed in 4% paraformaldehyde (PFA), and then adequately decalcified. Then, the femurs were sliced into 4 µm to 7 µm sections longitudinally. After that, femur sections were stained with hematoxylin and eosin, and digital pictures of trabecular bones were acquired by Olympus optical inverted microscope (Olympus, Tokyo, Japan).

### 2.4. Shallow Shotgun Sequencing

We extracted microbial DNA by QIAamp DNA Stool Mini Kit (QIAGEN, Germantown, MD, USA) from the fecal samples. Then, microbial DNA was fragmented into 400 bp reads before being constructed by NEXTflex™ DNA Sequencing Kit compatible with the Biomek^®^ FXp (Bio Scientific, Phoenix, AZ, USA) for shallow shotgun sequencing. After cluster generation, we generated the 2 × 300 base pairs (bp) paired-end reads on the Illumina Hiseq^TM^2500 platform. Then, Raw FASTQ files were filtered using FASTX-Tool kit. All reads were spliced after filtration, and then were assembled to form contigs using Mothur. According to the contigs sequence, we constructed scaffolds and set the main splicing parameter Kmer value to 55–85. All the scaffolds of more than 500 bp were counted for bioinformatics [24].

### 2.5. Bioinformatics

The sequence was trimmed to a quality score of more than 20, and the trimmed sequence was shorter than 80 bases. We mapped query reads against representative and reference genomes from the RefSeq database (version 82) using a 95% identity threshold. Then, we labeled reads that mapped to reference genomes with the NCBI taxonomic annotation. All reads that mapped to multiple reference genomes were labeled as the last common ancestor (LCA) of each label, and only species-level assignments are retained. To agree for species annotation, we used confidence-adjusted LCA that needs at least 80% of all tied best matches. α diversity indexes (ACE, Chao) were analyzed by Mothur (version 1.44.1). The commands are as follows: ACE estimator (http://www.mothur.org/wiki/Ace (accessed on 21 August 2020)), Chao1 estimator (http://www.mothur.org/wiki/Chao (accessed on 21 August 2020)). We performed principal component analysis (PCA) to visualize the ecological distance of the two groups. Microbial taxonomy was annotated and calculated by MEtaGenome Analyzer (MEGAN, version 5.2.3). Heatmaps were created by the Pheatmap package (version 1.0.12) of R Software. We combined Kruskal–Wallis test with linear discriminant analysis (LDA) value for LEfSe analysis. LEfSe analysis was performed based on two situations: an α significance level of 0.05 and threshold on the logarithmic LDA score for discriminative features equaling to 2.0. Based on KEGG ontology group (KO) annotation, we performed functional analysis of RefSeq derived genes in exhaustive gap alignment directly observed in shallow shotgun sequencing [25].

### 2.6. Statistical Analysis

We performed two independent-sample *t*-tests to analyze the statistical differences in relative abundances between the two groups by Graphpad prism (version 9.0) and STAMP. *p* < 0.05 was considered statistically significant. The significant difference of functional metabolic pathway abundances was defined by FDR-corrected *p*-value.

## 3. Results

### 3.1. Aged Rats Had Markedly Decreased BMD and Deteriorated Bone Microstructure

**Figure 1:**
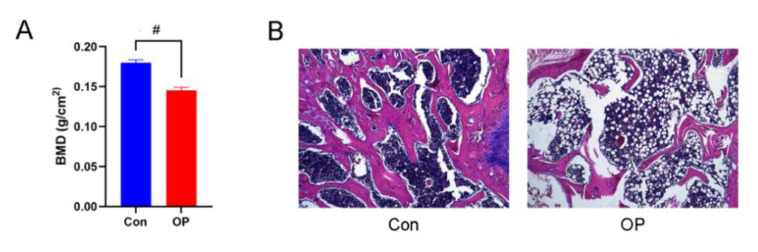
Aged rats had markedly decreased BMD and deteriorated bone microstructure: (**A**) The senile osteoporotic group had markedly lower BMD than the control group. # *p* < 0.01. (**B**) HE staining showed the femoral section in senile osteoporotic group had narrower trabecula thickness and fewer trabeculae number.

The BMD of 22-month old rats in the OP group was markedly lower than the control group (*p* < 0.01; Figure 1A). Moreover, as revealed by HE staining, the trabecula thickness was narrower and the trabeculae were fewer in the OP group. (Figure 1B). These results proved that aged rats had markedly decreased BMD and deteriorated bone microstructure.

### 3.2. Senile Osteoporotic Rats Showed the Altered Microbial Structure

**Figure 2:**
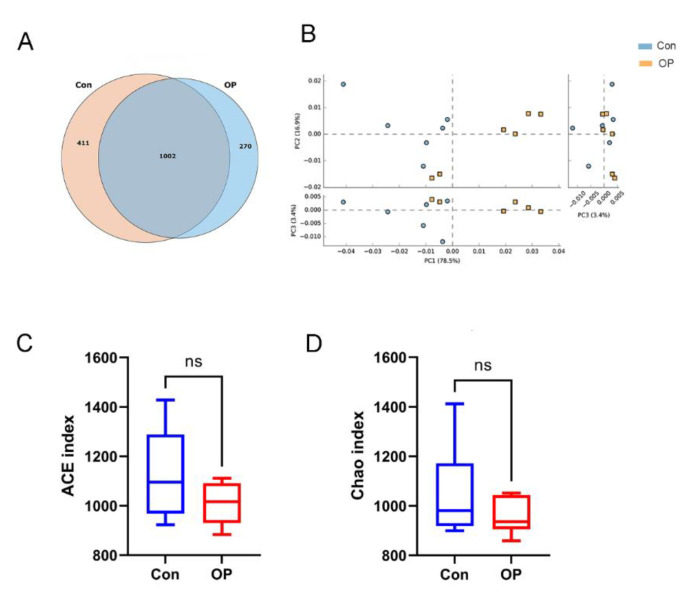
Senile osteoporotic rats showed the altered microbial structure: (**A**) Comparison of species numbers by Venn diagram; (**B**) PCA showed distinct β diversity between the OP group and the control group. (**C**) ACE indexes were decreased in the OP group. (**D**) Chao indexes were decreased in the OP group (ns: *p* > 0.05, no significance).

The results of the Venn diagram showed that the species number was lower in the OP group (1272) than in the control group (1413). The GM shared 1002 species between the two groups. A total of 411 species were quantified only in the control group, and 270 species were quantified only in the OP group (Figure 2A). PCA analysis was performed to calculate the ecological distance to analyze the β diversity of the samples in the two groups. PCA showed that samples in the control group (*n* = 6) and the OP group (*n* = 6) were well clustered and could be distinguished in each coordinate (Figure 2B). The results of PCA demonstrated that the structure of GM in senile osteoporotic rats was distinct from the young rats. The results of α diversity showed that ACE and Chao indexes of the OP group were lower than those in the control group (no significance; Figure 2C,D).

### 3.3. Senile Osteoporotic Rats Showed the Altered Microbial Component at the Genus Level

**Figure 3:**
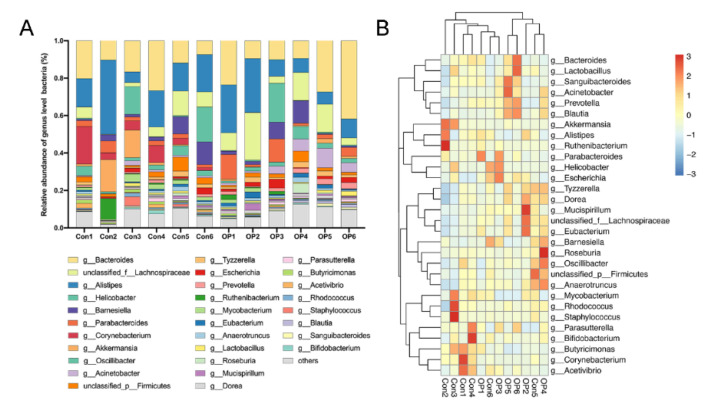
Senile osteoporotic rats showed the altered microbial component at the genus level: (**A**) Relative abundance of GM at the genus level. (**B**) Heat map revealed the differentially enriched genus.

At the genus level, the percentage stacked column chart showed that the relative abundance of *Bacteroide*, *Parabacteroides*, *Escherichia*, and *Prevotella* were more abundant in the OP group, while *Corynebacterium* and *Akkermansia* were more abundant in the control group (Figure 3A). Furthermore, the heatmap revealed that *Bacteroides*, *Lactobacillus*, *Sanguibacteroides*, *Acinetobacter*, *Prevotella*, and *Blautia* were enriched in the OP group. *Akkermansia*, *Corynebacterium*, and *Butryricimonas* were enriched in the control group (Figure 3B).

### 3.4. Senile Osteoporotic Rats Showed the Altered Microbial Component at the Species Level

**Figure 4:**
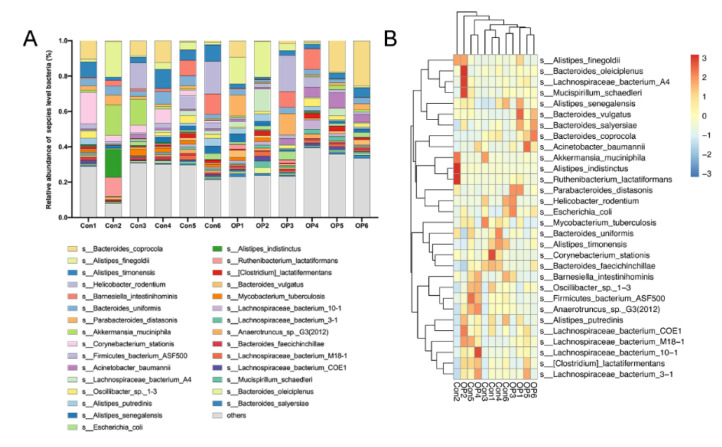
Senile osteoporotic rats showed the altered microbial component at the species level: (**A**) Relative abundance of GM at the species level. (**B**) Heat map revealed the differentially enriched species.

At the species level, the relative abundance analysis showed that *B. coprocola*, *A. baumannii*, *P. distasonis*, and *Lachnospiraceae bacterium* A4 were more abundant in the OP group, while *C. stationis*, *A. muciniphila*, and *A. indistinctus* were more abundant in the control group (Figure 4A). The heatmap showed that *B. coprocola*, *Lachnospiraceae bacterium* A4, and Bacteroides vulgatus were enriched in the OP group. *C. stationis* and *A. muciniphila* were enriched in the control group (Figure 4B).

### 3.5. Significantly Enriched GM at the Species Level

**Figure 5:**
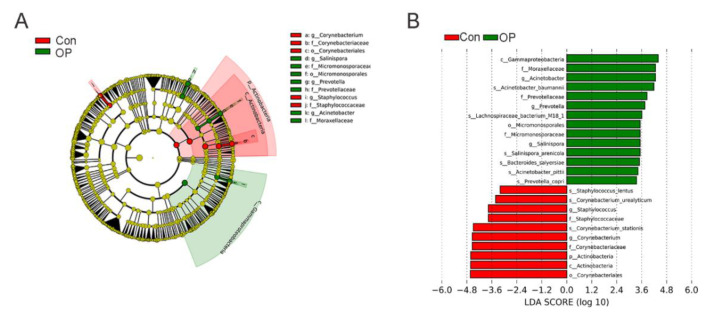
Linear discriminant effect size (LEfSe) analysis in senile osteoporotic group and the control group: (**A**) The evolutionary bifurcation graph showed that the abundance of *Prevotella* of *Prevotellaceae* family and *Acinetobacter* of Moraxellaceae family were enriched in the senile osteoporotic group. (**B**) LDA value distribution histogram showed that *Prevotella*, *Acinetobacter,* and *Salinispora* at the genus level and *A. baumannii*, *Lachnospiraceae_bacterium_M18_1*, and *P. copri* at the species level were enriched in the senile osteoporotic group.

We performed LEfSe analysis to ascertain the markedly enriched GM at the species level. The LEfSe evolutionary branching graph indicated that the abundance of *Prevotella* of *Prevotellaceae* family and Acinetobacter of *Moraxellaceae* family were markedly more abundant in the OP group (Figure 5A). Furthermore, LDA value distribution histogram showed that *Prevotella*, *Acinetobacter,* and *Salinispora* at the genus level and *A. baumannii*, *Lachnospiraceae bacterium M18 1*, and *P. copri* at the species level were significantly increased in the OP group (Figure 5B).

### 3.6. Significant Differences in the Functional Metabolic Pathways

**Figure 6:**
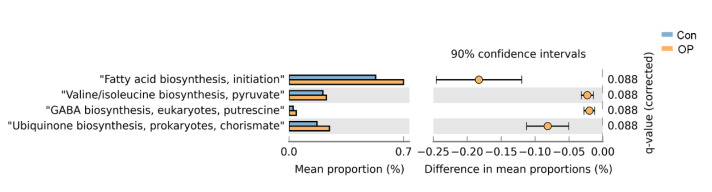
Senile osteoporotic rats had significantly different expressed functional pathways: fatty acid biosynthesis, Valine/isoleucine biosynthesis, GABA biosynthesis, and ubiquinone biosynthesis were more abundant in the senile osteoporotic group.

We found four significantly different expressed KEGG metabolic pathways between the two groups (all corrected *p* < 0.05). Metabolic pathways of fatty acid biosynthesis, Valine/isoleucine biosynthesis, GABA biosynthesis, and ubiquinone biosynthesis were significantly expressed in the OP group (Figure 6). 

## 4. Discussion

To the best of our knowledge, our study revealed the complete species-level GM information in senile osteoporosis for the first time. Using shallow shotgun sequencing technology, we found that the senile osteoporotic rats showed decreased species numbers, distinct β diversity, and low α diversity. Furthermore, the senile osteoporotic rats had markedly distinct GM compositions at the genus and species levels. At the genus level, *Prevotella*, *Acinetobacter,* and *Salinispora* significantly increased in osteoporotic group. At the species level, *A. baumannii*, *Lachnospiraceae bacterium M18 1*, and *P. copri* significantly increased in the osteoporotic group. In addition, KEGG functional pathways analysis found that fatty acid biosynthesis, Valine/isoleucine biosynthesis, GABA biosynthesis, and ubiquinone biosynthesis were enriched in the osteoporotic rats.

The GM structure was markedly changed between senile osteoporotic rats and young rats, which could be revealed by the distinct ecological distance in β diversity. Furthermore, the OP group had fewer unique species numbers than the control group, reflecting a decreased GM richness at the species level in the OP group. Several studies reported that disease statuses were related to decreased α diversity, such as type II diabetes and Alzheimer’s disease [26,27]. In our study, though there was no significant difference, ACE and Chao indexes suggested that the senile osteoporotic rats had lower α diversity, which was consistent with the previous study [17]. These results suggested that the species-level GM structure and richness markedly changed in the senile osteoporotic rats.

The relative abundance of GM could reflect the GM composition at the different taxonomic levels. At the genus level, the proportions of *Bacteroide*, *Parabacteroides*, *Escherichia*, and *Prevotella* were higher in the osteoporotic group, while the proportions of *Corynebacterium* and *Akkermansia* were higher in the control group. The increased proportion of *Prevotella* was consistent with the previous study [19]. At the species level, the proportion of *B. coprocola*, *A. baumannii*, *P. distasonis*, and *Lachnospiraceae bacterium A4* were higher in the OP group, while *C. stationis*, *A. muciniphila*, and *A. indistinctus* were more abundant in the control group. To identify significantly different GM, we performed LEfSe analysis and found that *Prevotella*, *Acinetobacter,* and *Salinispora* at the genus level and *A. baumannii*, *Lachnospiraceae bacterium M18 1*, and *P. copri* at the species level were enriched in the osteoporotic group. These results suggested that altered GM species were in accordance with the alteration of GM genus. Some GM species such as *P. copri* and *A. muciniphila* were proved to be linked to bone metabolism.

*P. copri*, which belongs to the *Prevotella* genus that also increased, was associated with a number of autoimmune diseases, such as colitis and rheumatoid arthritis [28,29,30]. Furthermore, *P. copri* was correlated with an increase in trimethylamine oxide (TMAO), a byproduct caused by dietary choline, which has an impact on cardiovascular disease and chronic kidney disease [31]. In addition, an increased level of TMAO has a negative correlation with the degree of bone mineral density (BMD) in osteoporosis patients [32]. In our work, *P. copri* was also found to be significantly enriched in the senile osteoporotic rats. *A. muciniphila* is a newly identified beneficial species in the phylum *Verrucomicrobia*. Previous studies reported that the abundance of *A. muciniphila* was reduced in aged humans and mice [33,34]. In an animal study, *A. muciniphila* was found to restore the bone mass of osteoporotic mice [35]. Consistently, we identified that *A. muciniphila* was decreased in the senile osteoporotic rats. Therefore, we suggested that the increased abundance of *P. copri* and decreased amount of *A. muciniphila* were closely related to the pathogenesis of senile osteoporosis. Furthermore, *A. baumannii,* which belongs to the *Acinetobacter genus,* is a major pathogenic factor of nosocomial infections [36]. *Salinispora* is a marine actinomycete genus that produces structurally diverse and biologically active secondary metabolites [37]. *Lachnospiraceae bacterium M18* and *Lachnospiraceae bacterium A4* belong to the Lachnospiraceae family, which could produce short-chain fatty acids and were beneficial for bone health [38]. The increase in these bacteria in aged rats could be attributed to the dysbiosis of gut microbiota. However, the association of these bacteria with the pathogenesis of senile osteoporosis needs further study.

KEGG functional pathway analysis found that metabolic pathways of fatty acid biosynthesis, Valine/isoleucine biosynthesis, GABA biosynthesis, and ubiquinone biosynthesis were enriched in the senile osteoporotic rats. Fatty acids metabolism and oxidative stress and production of ROS may impact each other and further influence bone metabolism [39]. As reported, oxidative stress was closely associated with aging [40]. Therefore, the enrichment of fatty acids biosynthesis might be related to aging-induced oxidative stress. Furthermore, GABA treatment could positively regulate osteogenic differentiation [41]. The enriched GABA biosynthesis in senile osteoporotic rats might be due to a compensatory effect after bone loss. These results suggested that fatty acid biosynthesis, Valine/isoleucine biosynthesis, GABA biosynthesis, and ubiquinone biosynthesis were related to senile osteoporosis.

## 5. Conclusions

We first manifested the complete information of species-level GM in senile osteoporotic rats. GM was significantly altered in structure and composition in senile osteoporotic rats. *B. coprocola*, *A. baumannii*, *P. distasonis*, and *Lachnospiraceae bacterium A4* and *P. copri* were higher in the senile osteoporotic group, while *C. stationis*, *A. muciniphila*, and *A. indistinctus* were decreased. Furthermore, KEGG function analysis revealed that metabolic pathways of fatty acid biosynthesis, Valine/isoleucine biosynthesis, GABA biosynthesis, and ubiquinone biosynthesis were enriched in the senile osteoporotic rats.

## Data Availability

The dataset generated during the current study are available from the corresponding author on reasonable request.
